# Medical student changes in self-regulated learning during the transition to the clinical environment

**DOI:** 10.1186/s12909-017-0902-7

**Published:** 2017-03-21

**Authors:** Kenneth K. Cho, Brahm Marjadi, Vicki Langendyk, Wendy Hu

**Affiliations:** School of Medicine, Western Sydney University, Campbelltown, NSW 2560 Australia

**Keywords:** Self-regulated learning, Clinical transition, Clerkship, Extrinsic motivation, Metacognition

## Abstract

**Background:**

Self-regulated learning (SRL), which is learners’ ability to proactively select and use different strategies to reach learning goals, is associated with academic and clinical success and life-long learning. SRL does not develop automatically in the clinical environment and its development during the preclinical to clinical learning transition has not been quantitatively studied. Our study aims to fill this gap by measuring SRL in medical students during the transitional period and examining its contributing factors.

**Methods:**

Medical students were invited to complete a questionnaire at the commencement of their first clinical year (T0), and 10 weeks later (T1). The questionnaire included the Motivated Strategies for Learning Questionnaire (MSLQ) and asked about previous clinical experience. Information about the student’s background, demographic characteristics and first clinical rotation were also gathered.

**Results:**

Of 118 students invited to participate, complete paired responses were obtained from 72 medical students (response rate 61%). At T1, extrinsic goal orientation increased and was associated with gender (males were more likely to increase extrinsic goal orientation) and type of first attachment (critical care and community based attachments, compared to hospital ward based attachments). Metacognitive self-regulation decreased at T1 and was negatively associated with previous clinical experience.

**Conclusions:**

Measurable changes in self-regulated learning occur during the transition from preclinical learning to clinical immersion, particularly in the domains of extrinsic goal orientation and metacognitive self–regulation. Self–determination theory offers possible explanations for this finding which have practical implications and point the way to future research. In addition, interventions to promote metacognition before the clinical immersion may assist in preserving SRL during the transition and thus promote life-long learning skills in preparation for real-world practice.

**Electronic supplementary material:**

The online version of this article (doi:10.1186/s12909-017-0902-7) contains supplementary material, which is available to authorized users.

## Background

In the context of a constantly advancing medical world, the medical profession must consistently meet the high standards of optimal patient care [1, 2] and maintain their area of expertise throughout their careers [3, 4]. It is important that doctors become life-long learners who take control of their learning needs and activities through self-regulated learning [1, 2, 5]. Self-regulated learning (SRL) is defined as a process where the learner is motivationally, behaviourally and meta-cognitively proactive in the learning process [5]. In the clinical environment SRL has been associated with academic achievement [4, 6–8], success in clinical skills [9] and emotional health [10].

The initial transition from preclinical learning to clinical immersion is a significant and unique phase in a medical student’s education when students shift from spending more time learning in the classroom to experiential learning in the clinical setting [11–15]. In contrast to preclinical learning, students in immersive clinical rotations report the elements of adjusting to new environments with heavy workloads and long working hours [12, 16, 17], adapting to different expectations, forms of assessment and teaching styles [12, 18], feeling at times useless and uncertain about their role [12, 19, 20] and adapting to a more self–directed learning style [16, 20, 21]. Specifically, research suggests that despite the addition of clinical tutorials to the preclinical curriculum, students during the clinical transition face considerable challenges, ambiguity and uncertainty [12, 22, 23]. As well as opportunities, these experiences present potential disruptions to the development of the skills needed for life-long learning. However to our knowledge, no research has explored the changes in self -regulated learning that occur during the initial transition period from preclinical to clinical immersion using a quantitative approach.

We therefore aimed to measure the changes that occur in self-regulated learning during the critical transition from pre-clinical to immersive clinical learning and associated factors. We hypothesized that changes in SRL would occur, as the learning environment is a key factor affecting SRL in many theoretical models [2, 24, 25]. Our research questions were: “what are the changes in SRL during the transition to the clinical learning environment?” and “what factors are associated with that change?” Through these research questions we aimed to enhance our understanding of medical student motivation and learning during the clinical transitional period, which has implications on curricula and future research directions.

## Method

### Study setting

The study participants were 3rd year medical students at Western Sydney University, Australia. The curriculum is a 5-year undergraduate program. The first 2 years comprise of problem based learning in small groups with weekly clinical tutorials in hospital settings. The final 3 years are based on a model of clinical immersion in rural and metropolitan hospitals as well as community based attachments. In the first clinical year (3rd year of the course) there are seven rotations in a year, where each rotation is of a 5-week duration: two medical, two surgical, two community (consisting of community health service and general practice) and one critical care (consisting of emergency department and anaesthetics) attachments.

### Study participants

This study was conducted in 2015 with a cohort of students (*n* = 118) at the beginning of first clinical year. Ethical approval was obtained from Western Sydney University (ID H9989).

#### Data collection

Consenting students were invited to complete a questionnaire at the introductory lectures prior to the commencement of their first clinical placement (Time 0, T0). The survey was repeated after the first 10 weeks of clinical placements (Time 1, T1). Only those who responded at both T0 and T1 and where paired responses could be identified were included in the analysis. The T1 time of 10 weeks was chosen as the organizational psychology literature suggests newcomers learn, adjust and adapt to the job, roles and culture of a workplace rapidly in the first 1–2 months post-entry period [26–29], that the resulting adjustments are relatively stable [30–32] and that early adaptations are related to important outcomes for both organizations and new employees [29, 30, 33, 34].

### Motivated Strategies for Learning Questionnaire (MSLQ)

The MSLQ is a validated instrument based upon the social cognitive theory of learning to measure SRL [35, 36]. The instrument has been used in a wide range of population groups, from students in primary schools to those in higher education. Several studies have used the MSLQ as part of medical education research and have found associations between MSLQ scores and academic achievement [6, 37–39]. One preliminary study used the MSLQ to measure the changes in SRL of preclinical medical students that occurred between the first and second year of their course [40].

The MSLQ uses a 7-point Likert-type scale comprising of 2 sections, motivation and learning strategies. The motivation section contains 31 items assessing 3 domains: goal orientation, self-belief about learning, and test-provoked anxiety. The learning section contains 50 items assessing 3 domains: the use of cognitive strategies, metacognitive strategies and resource management (Additional file 1: Appendix 1).

### First clinical rotation

Data was extracted from student records and included information about what the students’ first clinical attachment would be: medicine, surgery, critical care or the community attachment.

### Participant background

Demographic data such as age, gender and entry status were extracted from student records. Entry status categories included school-leaver students who entered immediately after high school; non school-leaver students who started but have not finished another tertiary degree; graduate students who have completed another tertiary degree; and international students. As part of the T0 questionnaire, students were asked whether they had any prior clinical experience other than the compulsory clinical medicine tutorials during the first 2 years.

#### Data analysis

For each subscale of the MSLQ, the data were categorized into 3 categories: low scores (1.0 to <2.5), medium scores (2.5 to <5), and high scores (5 to <7). The Marginal Homogeneity Test was used to assess the significance of differences in each MSLQ subscale between T0 and T1. For subscales which were found to have a significant difference (*p* < 0.05), respondents’ scores were categorised as having increased, decreased or stayed in the same categories between T0 and T1. Ordinal Logistic Regression and Multiple Logistic Regression were then used to explore which dependent variables influenced the MSLQ subscale score changes between T0 and T1. As age reflects entry status, and entry status was considered more relevant to SRL than age, only entry status was entered into the regression analyses to avoid multi-collinearity. In the Ordinal Logistic Regression, the proportionality-of-odds assumption was evaluated by the likelihood-ratio test. All statistical analysis was performed with IBM SPSS Statistics 22.

For the purposes of analysis, medical and surgical first attachment groups were then combined. The rationale for this is that both medical and surgical attachments are similar in structure with 1–2 students being attached to relatively large ward-based teams and have the same clinical assessments. The critical care attachment does not involve ward round-based teaching but instead 1:1 shift-based clinical supervision with more formal tutorials. The community attachment also involves 1:1 supervision but focuses less on disease and treatment and more on the psychosocial, cultural, environmental and economic elements that can affect health.

## Results

Of 118 students who were invited to participate, 94 responses were obtained at T0 (response rate 80%), and 75 responses were obtained at T1 (response rate 64%). Paired, complete responses were obtained from 72 medical students (response rate 61%).

Of the 72 respondents, most were female (61.1%), non-school leavers (44.4%), had previous clinical exposure (51.4%) and their first attachment was either medicine or surgery (58.3%). The mean age of respondents was 21.3 years (range 19–30, standard deviation, 1.7; Table 1). There were no statistically significant difference between the sample and the whole cohort with regard to gender, age, entry status or first rotation (all *p* > 0.05; data not shown).

**Table 1:** Characteristics of survey respondents

Total Participants	Number	Percentage (%)
Gender		
Male	28	38.9
Female	44	61.1
Entry Status		
School leaver	25	34.7
Non-school leaver	32	44.4
Graduate student	3	4.2
International student	12	16.7
Previous Exposure		
No previous clinical exposure	35	48.6
Previous clinical exposure	37	51.4
First Attachment		
Medicine or surgery	42	58.3
Critical Care	8	11.1
Community attachment	22	30.6

Table 2 shows the change in SRL measured by the MSLQ between T0 and T1. Two scales (extrinsic goal orientation and metacognitive self-regulation) in the MSLQ showed significant differences between T0 and T1 (*p* < 0.033, *p* < 0.001 respectively). Extrinsic motivation increased and metacognitive self-regulation decreased. Domains of self-regulated learning that did not change in our study included the motivation scales of intrinsic goal orientation, task value, control beliefs, self-efficacy and test anxiety as well as the learning strategy scales of rehearsal, elaboration, organization, critical thinking, time and study environment regulation, effort regulation, peer learning and help-seeking behaviour.

**Table 2:** Change in the MSLQ from T_0_ to T_1_

Section	Subscale	Number of participants, T0			Number of participants, T1			*P* values of T0-T1 difference
		Low score	Medium score	High score	Low score	Medium score	High score	
Motivation	Intrinsic Goal Orientation	0	27	45	0	19	53	0.061
	Extrinsic Goal Orientation	1	45	26	1	35	36	0.033^a^
	Task Value	0	5	67	0	8	64	0.180
	Control of Learning Beliefs	0	14	58	0	15	57	0.782
	Self-Efficacy for Learning and Performance	1	42	29	1	37	34	0.275
	Test Anxiety	3	46	23	4	43	25	0.827
Learning Strategies	Rehearsal	1	58	13	3	46	23	0.074
	Elaboration	0	29	43	0	32	40	0.532
	Organization	0	36	36	0	37	35	0.827
	Critical Thinking	2	58	12	3	52	17	0.317
	Metacognitive Self-regulation	0	54	18	0	66	6	0.001^b^
Resource Management Strategies	Time and Study Environment	1	47	24	0	53	19	0.317
	Effort Regulation	1	48	23	0	47	25	0.532
	Peer Learning	9	47	16	7	42	23	0.072
	Help Seeking	5	51	16	4	51	17	0.655

^a^Statistically significant at the 0.05 level (Marginal Homogeneity Test)

^b^Statistically significant at the 0.01 level (Marginal Homogeneity Test)

The factors “entry status”, “gender”, “previous clinical experience” and “first clinical attachment” were entered into an ordinal logistic regression for extrinsic goal orientation. For metacognitive self-regulation as the data distribution was dichotomous, a binary logistic regression was used. Both regression analyses are summarized in Table 3. Regarding extrinsic goal orientation, two independent factors were identified: gender and first attachment. Male students were more likely to increase in extrinsic goal orientation (OR 4.1, 95% CI 1.2–13.5, *p* < 0.021) as were students on critical care (OR 8.7, 95% CI 1.6–48.5, *p* < 0.013) and students on the community attachment (OR 3.8, 95% CI 1.1–14.0, *p* < 0.042). Concerning metacognitive self-regulation the sole independent factor was previous clinical experience—students with extra previous clinical experience were more likely to have lower levels of metacognitive self-regulation (OR 5.0 95% CI 1.1–22.2, *p* < 0.035).

**Table 3:** Results of Regression Analysis

Domain	Factors	Category	Odds Ratio (95% CI)	*P* value
Extrinsic Goal Orientation^a^				
	Gender	Male vs Female	4.1 (1.234–13.526)	0.021
	First Attachment	Critical Care vs Medicine/Surgery	8.7 (1.570–48.543)	0.013
	First Attachment	Community attachment vs Medicine/Surgery	3.8 (1.052–14.015)	0.042
Metacognitive Self-Regulation^b^				
	Previous clinical experience	Experience vs No experience	5.0 (1.123–22.170)	0.035

^a^Non-significant factors in the model were “entry status” and “previous clinical experience”

^b^Non-significant factors in the model were “entry status”, “gender” and “first clinical attachment

## Discussion

This study has investigated the changes in SRL during the transition to clinical learning in the first clinical year and identified factors associated with that change. Our results indicated changes occurred in the two domains of extrinsic goal orientation and metacognitive self-regulation. Factors associated with an increase in extrinsic goal orientation were gender (male vs female) and first attachment (critical care and the community attachment vs medicine/surgery). The single factor associated with a decrease in metacognitive self-regulation was previous clinical experience (no experience vs experience).

### Extrinsic motivation

According to the MSLQ, extrinsic goal orientation is defined as the degree to which a student perceives the importance of issues that are not directly related to participating in the task itself [41]. This includes grades, rewards and reputation. Studies from the transitions literature provide an explanation as to why transitioning clerkship students increase in extrinsic motivation, and how this may relate to their gender and first attachment.

As opposed to the preclinical years where students are assessed in formal examination settings, the literature suggests students in immersive clinical settings feel constantly informally assessed by their supervisors. [42–46]. These studies suggest clerkship students are more extrinsically motivated in their learning as impressing their supervisor may lead to further experiential learning opportunities, better evaluations and future career prospects [42–46]. This also explains why in our study, compared to the transitioning students on the ward-based attachments, the students whose first rotations involved 1:1 supervision (the critical care and the community attachments) were more likely to increase in extrinsic goal orientation.

In regards to gender, our study found that male students are more likely to develop an increase in extrinsic goal orientation with qualitative studies from the transitions literature supporting our finding [47, 48]. Supervisors may have gender-biased expectations of performance which lead to female medical students receiving less pressure [47].

A more nuanced explanation as to why students experience an increase in extrinsic goal orientation during the transition to the clinical environment can be provided by the self-determination theory (SDT) of Ryan and Deci, which is a more recent model of motivation to the MSLQ. Researchers widely agree that when possible, intrinsic motivation is preferred as it has been linked with more enjoyment [49], more engagement [50, 51] and better learning [52, 53]. According to Ryan and Deci, for extrinsic motivation to develop into intrinsic motivation, the student must be interested in the task at hand as well as have their needs of competence, autonomy and relatedness met [54]. According to SDT, competence refers to the experience of behaviour being effectively enacted. The feeling of competence is supported when activities are optimally challenging, thereby allowing students to test and expand their academic capabilities, or when feedback promotes feelings of efficacy or eventual mastery [49]. Autonomy occurs when a student’s behaviour is aligned with their authentic interests or integrated values and desires, and when the student fully endorses the actions they engaged in or the values they expressed [53, 55]. Autonomy is lost when the student feels they are compelled to behave in specific ways regardless of their own values or interests [55]. The need for relatedness refers to the tendency for people to internalise and adopt the values and the practices of those they feel connected to or desire a connection with, and from contexts where they feel belonging [49]. Relatedness is supported when a teacher genuinely likes, respects and values the student [49]. According to SDT, if the needs of competence, autonomy and relatedness are not met, any motivation will be extrinsic as opposed to intrinsic in nature.

When analysed through the frame of SDT, previous studies in the transitions literature suggests medical students in the transition period may experience a lack of all three needs: students feel they lack competence, autonomy and relatedness [11, 16, 46, 56–59]. Radcliffe and Lester found that during the transition students had experiences “of feeling useless [and] unable to contribute to patient care because they had insufficient knowledge or skills” [lack of competence] [11]. The lack of competence felt by medical students during the clinical transition is supported by other studies [58, 59]. In regards to autonomy, studies suggest that students feel they often complete tedious tasks such as paperwork at the request of their consultant physician instead of engaging in tasks more aligned with their interests and values, such as talking to patients [16, 46]. In regards to relatedness, students may feel they are neither being genuinely valued nor respected, with older studies suggesting that levels of abuse (verbal, physical, sexual and academic) experienced by medical students are high (50–93%) [56, 60–62].

Newer research suggest some forms of extrinsic motivation may be similar to intrinsic motivation [53]. It is important to state that there are—according to self-determination theory—4 types of extrinsic motivation (externally regulated, introjected, identified and integrated). Externally regulated motivation (where behaviours are enacted to obtain a reward or to avoid punishment) and introjected motivation (whereby behaviours are enacted in order to primarily protect one’s ego) are believed to be shallower forms of motivation whose behaviours are poorly maintained once the controlling extrinsic factors have been removed [54]. Identified motivation (where behaviours are enacted because of the perceived value of the task) and integrated motivation (where behaviours are enacted because they are aligned with other aspects of self) are believed to be deeper forms of motivation, whose behaviour stems from more autonomous motivation [53, 54]. The distinction between the former two and latter two types of motivations is critical because studies in educational psychology suggest that higher amounts of autonomous extrinsic motivation are linked with academic success, quicker adjustment, greater well-being, decreased anxiety and more intrinsic enjoyment [49, 63–65] which are all highly relevant to the transitions period. Thus it may be well worth for the MSLQ extrinsic motivation subscale to be revised so that it can distinguish between each of the different types of extrinsic motivation. Practically this is important, because if the increases in extrinsic motivation are not beneficial, then curriculum designers could structure first attachments so that transitioning students feel less monitored and more autonomous in their learning.

### Metacognitive self-regulation

The MSLQ defines metacognition as the awareness and control of cognition that can be broken down into three general processes: planning, monitoring, and regulating [41]. Planning activities include goal setting and reflecting on prior knowledge that make organizing and comprehending the material easier. Monitoring activities include the tracking of attention and self-testing. Regulating activities include adjusting one’s cognitive and behavioural activities.

In our study, previous clinical experience was associated with a decrease in metacognitive self-regulation. This finding was surprising as we hypothesised that students with previous clinical experience would find the transition period less stressful and thus need to use less cognitive resources to adapt, spending more of their cognitive resources on metacognition. These hypotheses are consistent with previous transitions literature suggesting that prior clinical experience leads to a smoother transition [66–68] and potentially less cognitive load [69]. There is no clear reason for our findings from this research.

The decrease in metacognitive self-regulation has real-world importance. Studies suggest that metacognition has a positive association with academic performance [35] and surgical skills acquisition [70], a negative association with procrastination [4] and depression [9], and is important for clinical reasoning, decision making [71, 72] and the continuous process of life-long learning [73, 74]. Furthermore, positive metacognitive abilities have been associated with a decreased level of perceived stress [75]. On a conceptual level, because clerkships are based upon the principles of experiential learning, the success of clerkship depends in part on a student’s capacity for reflective practice and accurate self-assessment [76, 77]. Therefore metacognitive self-regulation is critical for students to be able to learn effectively, especially during the immersive clinical years as studies suggest that the student interactions with patients are rarely observed directly by clinical teachers [78, 79].

Fortunately, literature suggests that interventions can increase metacognitive processes. Chew showed a simple metacognitive checklist could facilitate metacognition in clinical decision-making [80], Sobral showed that a 30 h learning skills course for medical students could increase levels of reflection, one subset of metacognition [7] and Tanner suggests explicitly teaching metacognition may be efficacious [81]. Within the hospital, studies also suggest supervisors can increase the metacognition of their students by providing feedback [82], by “thinking-aloud”—which involves vocalizing their thought processes involved in clinical reasoning [83] and by emphasizing the importance of learning over outcome [84]. Our research suggests a metacognitive intervention before or during the transition may be valuable so that students can experience less stress and optimize their learning.

### Limitations

Our study had several limitations. Due to the single-institution design of the study, care must be taken not to over interpret our findings particularly with respect to transitions in different medical schools. Furthermore our study focussed on a single transition and thus transferability to other cohorts is limited. However, as the clinical transition structure is the same across the years, it is possible that similar trends may exist in other cohorts. A questionnaire was our main data collection tool, therefore social desirability bias may be present. However the MSLQ has reasonable psychometric properties [34]. With our factor “first attachment” there is likely to be inherent differences within rotations. For example, two medical rotations could have different supervisor–student dynamics. Despite this likely diversity of experiences within the attachments, a significant effect of first attachment was still found for extrinsic motivation. The results of our study had wide confidence intervals and negative results and therefore a larger study should be conducted to get a clearer insight into the transitional period. Finally, due to the response rate we obtained, a possibility of selection bias and type 2 error exists.

## Conclusion

Our study explored the changes in the SRL of medical students during the transition to immersive clinical learning using a quantitative approach. We found that 10 weeks after transitioning to clinical learning, students significantly increased in extrinsic goal orientation and significantly decreased in metacognitive self-regulation. Factors associated with the increase in extrinsic goal orientation were gender and first clinical attachment, with the style of clinical supervision being a possible explanation for the observed differences between attachments. The sole factor associated with the decrease in metacognitive self-regulation was previous clinical experience. Although a larger study with multiple cohorts from multiple institutions is necessary to improve the generalizability of our findings, our study suggests that future research could further explore the transition to clinical learning through the lens of SDT, as well as interventions to enhance metacognition and thus learning during the transition period.

## Additional file

Additional file 1: Appendix 1 The Motivated Strategies for Learning Questionnaire (MSLQ). Description: The MSLQ, a validated instrument based upon the social cognitive theory of learning to measure SRL. (DOCX 52 kb)

## Abbreviations

MSLQ: Motivated strategies for learning questionnaire
SDT: Self determination theory
SRL: Self-regulated learning
