# Supplementary material for: Medical student changes in self-regulated learning during the transition to the clinical environment
Source: BMC Med Educ. 2017 Mar 21;17:59. doi: 10.1186/s12909-017-0902-7 (PMC5361773; doi:10.1186/s12909-017-0902-7)
Supplement: Additional file 1: — Appendix 1 The Motivated Strategies for Learning Questionnaire (MSLQ). Description: The MSLQ, a validated instrument based upon the social cognitive theory of learning to measure SRL. (DOCX 52 kb) [file 12909_2017_902_MOESM1_ESM.docx]

Additional Files Legend

-File name: Appendix 1
-Title of contents: Appendix 1 - The Motivated Strategies for Learning Questionnaire (MSLQ)
-Description: The MSLQ, a validated instrument based upon the social cognitive theory of learning to measure SRL.

**Additional file 1: Appendix 1 - The Motivated Strategies for Learning Questionnaire (MSLQ)**

**The Motivated Strategies for Learning Questionnaire**

Please rate the following items based on your current experience. Your rating should be on a 7 point scale where **1 = not at all true to me** and **7 = very true of me.**

|  | Please indicate how much you agree with the following statements: | **1** | **2** | **3** | **4** | **5** | **6** | **7** |
| --- | --- | --- | --- | --- | --- | --- | --- | --- |
|  | Part A: Motivation |  |  |  |  |  |  |  |
| 1) | In a class like this, I prefer course material that really challenges me so I can learn new things. |  |  |  |  |  |  |  |
| 2) | If I study in appropriate ways, then I will be able to learn the material in this course. |  |  |  |  |  |  |  |
| 3) | When I take a test I think about how poorly I am doing compared with other students. |  |  |  |  |  |  |  |
| 4) | I think I will be able to use what I learn in this course in other areas of my life. |  |  |  |  |  |  |  |
| 5) | I believe I will receive an excellent grade in this class. |  |  |  |  |  |  |  |
| 6) | I’m certain I can understand the most difficult material presented in the readings for this course. |  |  |  |  |  |  |  |
| 7) | Getting a good grade in this class is the most satisfying thing for me right now. |  |  |  |  |  |  |  |
| 8) | When I take a test I think about items on other parts of the test I can’t answer. |  |  |  |  |  |  |  |
| 9) | It is my own fault if I don’t learn the material in this course. |  |  |  |  |  |  |  |
| 10) | It is important for me to learn the course material in this class. |  |  |  |  |  |  |  |
| 11) | The most important thing for me right now is improving my overall grade point average, so my main concern in this class is getting a good grade. |  |  |  |  |  |  |  |
| 12) | I’m confident I can learn the basic concepts taught in this course. |  |  |  |  |  |  |  |
| 13) | If I can, I want to get better grades in this class than most of the other students. |  |  |  |  |  |  |  |
| 14) | When I take tests I think of the consequences of failing. |  |  |  |  |  |  |  |
| 15) | I’m confident I can understand the most complex material presented by the instructors in this course. |  |  |  |  |  |  |  |
| 16) | In a class like this, I prefer course material that arouses my curiosity, even if it is difficult to learn. |  |  |  |  |  |  |  |
| 17) | I am very interested in the content area of this course. |  |  |  |  |  |  |  |
| 18) | If I try hard enough, then I will understand the course material. |  |  |  |  |  |  |  |
| 19) | I have an uneasy, upset feeling when I take an exam. |  |  |  |  |  |  |  |
| 20) | I’m confident I can do an excellent job on the assignments and tests in this course. |  |  |  |  |  |  |  |
| 21) | I expect to do well in this class. |  |  |  |  |  |  |  |
| 22) | The most satisfying thing for me in this course is trying to understand the content as thoroughly as possible. |  |  |  |  |  |  |  |
| 23) | I think the course material in this class is useful for me to learn. |  |  |  |  |  |  |  |
| 24) | When I have the opportunity in this class, I choose course assignments that I can learn from even if they don’t guarantee a good grade. |  |  |  |  |  |  |  |
| 25) | If I don’t understand the course material, it is because I didn’t try hard enough. |  |  |  |  |  |  |  |
| 26) | I like the subject matter of this course. |  |  |  |  |  |  |  |
| 27) | Understanding the subject matter of this course is very important to me. |  |  |  |  |  |  |  |
| 28) | I feel my heart beating fast when I take an exam. |  |  |  |  |  |  |  |
| 29) | I’m certain I can master the skills being taught in this class. |  |  |  |  |  |  |  |
| 30) | I want to do well in this class because it is important to show my ability to my family, friends, employer, or others. |  |  |  |  |  |  |  |
| 31) | Considering the difficulty of this course, the teacher, and my skills, I think I will do well in this class. |  |  |  |  |  |  |  |
|  | **Part B: Learning Strategies** |  |  |  |  |  |  |  |
| 32) | When I study the readings for this course, I outline the material to help me organize my thoughts. |  |  |  |  |  |  |  |
| 33) | During class time I often miss important points because I’m thinking of other things. |  |  |  |  |  |  |  |
| 34) | When studying for this course, I often try to explain the material to a classmate or friend. |  |  |  |  |  |  |  |
| 35) | I usually study in a place where I can concentrate on my course work. |  |  |  |  |  |  |  |
| 36) | When reading for this course, I make up questions to help focus my reading. |  |  |  |  |  |  |  |
| 37) | I often feel so lazy or bored when I study for this class that I quit before I finish what I planned to do. |  |  |  |  |  |  |  |
| 38) | I often find myself questioning things I hear or read in this course to decide if I find them convincing. |  |  |  |  |  |  |  |
| 39) | When I study for this class, I practice saying the material to myself over and over. |  |  |  |  |  |  |  |
| 40) | Even if I have trouble learning the material in this class, I try to do the work on my own, without help from anyone. |  |  |  |  |  |  |  |
| 41) | When I become confused about something I’m reading for this class, I go back and try to figure it out. |  |  |  |  |  |  |  |
| 42) | When I study for this course, I go through the readings and my class notes and try to find the most important ideas. |  |  |  |  |  |  |  |
| 43) | I make good use of my study time for this course. |  |  |  |  |  |  |  |
| 44) | If course materials are difficult to understand, I change the way I read the material. |  |  |  |  |  |  |  |
| 45) | I try to work with other students from this class to complete the course assignments. |  |  |  |  |  |  |  |
| 46) | When studying for this course, I read my class notes and the course readings over and over again. |  |  |  |  |  |  |  |
| 47) | When a theory, interpretation, or conclusion is presented in class or in the readings, I try to decide if there is good supporting evidence. |  |  |  |  |  |  |  |
| 48) | I work hard to do well in this class even if I don’t like what we are doing. |  |  |  |  |  |  |  |
| 49) | I make simple charts, diagrams, or tables to help me organize course material. |  |  |  |  |  |  |  |
| 50) | When studying for this course, I often set aside time to discuss course material with a group of students from the class. |  |  |  |  |  |  |  |
| 51) | I treat the course material as a starting point and try to develop my own ideas about it. |  |  |  |  |  |  |  |
| 52) | I find it hard to stick to a study schedule. |  |  |  |  |  |  |  |
| 53) | When I study for this class, I pull together information from different sources, such as lectures, readings, and discussions. |  |  |  |  |  |  |  |
| 54) | Before I study new course material thoroughly, I often skim it to see how it is organized. |  |  |  |  |  |  |  |
| 55) | I ask myself questions to make sure I understand the material I have been studying in this class. |  |  |  |  |  |  |  |
| 56) | I try to change the way I study in order to fit the course requirements and the instructor’s teaching style. |  |  |  |  |  |  |  |
| 57) | I often find that I have been reading for this class but don’t know what it was all about. |  |  |  |  |  |  |  |
| 58) | I ask the instructor to clarify concepts I don’t understand well. |  |  |  |  |  |  |  |
| 59) | I memorize key words to remind me of important concepts in this class. |  |  |  |  |  |  |  |
| 60) | When course work is difficult, I either give up or only study the easy parts. |  |  |  |  |  |  |  |
| 61) | I try to think through a topic and decide what I am supposed to learn from it rather than just reading it over when studying for this course. |  |  |  |  |  |  |  |
| 62) | I try to relate ideas in this subject to those in other courses whenever possible. |  |  |  |  |  |  |  |
| 63) | When I study for this course, I go over my class notes and make an outline of important concepts. |  |  |  |  |  |  |  |
| 64) | When reading for this class, I try to relate the material to what I already know. |  |  |  |  |  |  |  |
| 65) | I have a regular place set aside for studying. |  |  |  |  |  |  |  |
| 66) | I try to play around with ideas of my own related to what I am learning in this course. |  |  |  |  |  |  |  |
| 67) | When I study for this course, I write brief summaries of the main ideas from the readings and my class notes. |  |  |  |  |  |  |  |
| 68) | When I can’t understand the material in this course, I ask another student in this class for help. |  |  |  |  |  |  |  |
| 69) | I try to understand the material in this class by making connections between the readings and the concepts from the lectures. |  |  |  |  |  |  |  |
| 70) | I make sure that I keep up with the weekly readings and assignments for this course. |  |  |  |  |  |  |  |
| 71) | Whenever I read or hear an assertion or conclusion in this class, I think about possible alternatives. |  |  |  |  |  |  |  |
| 72) | I make lists of important items for this course and memorize the lists. |  |  |  |  |  |  |  |
| 73) | I attend this class regularly. |  |  |  |  |  |  |  |
| 74) | Even when course materials are dull and uninteresting, I manage to keep working until I finish. |  |  |  |  |  |  |  |
| 75) | I try to identify students in this class whom I can ask for help if necessary. |  |  |  |  |  |  |  |
| 76) | When studying for this course I try to determine which concepts I don’t understand well. |  |  |  |  |  |  |  |
| 77) | I often find that I don’t spend very much time on this course because of other activities. |  |  |  |  |  |  |  |
| 78) | When I study for this class, I set goals for myself in order to direct my activities in each study period. |  |  |  |  |  |  |  |
| 79) | If I get confused taking notes in class, I make sure I sort it out afterwards. |  |  |  |  |  |  |  |
| 80) | I rarely find time to review my notes or readings before an exam. |  |  |  |  |  |  |  |
| 81) | I try to apply ideas from course readings in other class activities such as lecture and discussion. |  |  |  |  |  |  |  |
